# Short-term dietary changes are reflected in the cerebral content of adult ring-billed gulls

**DOI:** 10.1098/rsos.240616

**Published:** 2024-08-07

**Authors:** Jessika Lamarre, David R. Wilson

**Affiliations:** ^1^ Cognitive and Behavioural Ecology Program, Memorial University of Newfoundland, St John’s, Newfoundland and Labrador A1B 3X9, Canada; ^2^ Department of Psychology, Memorial University of Newfoundland, St John’s, Newfoundland and Labrador A1B 3X9, Canada

**Keywords:** omega-3 fatty acids, bird, brain plasticity, stable isotope, diet specialization, marine ecosystem

## Abstract

Omega-3 long-chain polyunsaturated fatty acids (n3-LCPUFAs) are produced primarily in aquatic ecosystems and are considered essential nutrients for predators given their structural role in vertebrates’ cerebral tissues. Alarmingly, with urbanization, many aquatic animals now rely on anthropogenic foods lacking n3-LCPUFAs. In this study undertaken in Newfoundland (Canada), we tested whether recent or longer term diet explains the cerebral fatty acid composition of ring-billed gulls (*Larus delawarensis*), a seabird that now thrives in cities. During the breeding season, cerebral levels of n3-LCPUFAs were significantly higher for gulls nesting in a natural habitat and foraging on marine food (mean ± s.d.: 32 ± 1% of total identified fatty acids) than for urban nesters exploiting rubbish (27 ± 1%). Stable isotope analysis of blood and feathers showed that urban and natural nesters shared similar diets in autumn and winter, suggesting that the difference in cerebral n3-LCPUFAs during the breeding season was owing to concomitant and transient differences in diet. We also experimentally manipulated gulls’ diets throughout incubation by supplementing them with fish oil rich in n3-LCPUFAs, a caloric control lacking n3-LCPUFAs, or nothing, and found evidence that fish oil increased urban nesters’ cerebral n3-LCPUFAs. These complementary analyses provide evidence that the brain of this seabird remains plastic during adulthood and responds to short-term dietary changes.

## 1. Introduction

Several species thrive in urban environments, in part, because they have access to abundant and consistent anthropogenic food resources [1–3]. Yet, anthropogenic foods often lack nutritional quality, potentially causing nutritional deficiencies in essential amino acids, fatty acids or micronutrients [4–6]. Western diets are notably deficient in omega-3 fatty acids (n3-PUFAs), which include the medium-chain alpha-linolenic acid (ALA) and its long-chain derivates omega-3 long-chain polyunsaturated fatty acids (n3-LCPUFAs), namely eicosapentaenoic acid (EPA), docosapentaenoic acid (DPA) and docosahexaenoic acid (DHA) [7,8]. These three n3-LCPUFAs are critical for brain development and maintenance in vertebrates [9–11]. DHA specifically is one of the most important structural components of neuronal tissue in vertebrates [10–13], and, in mammals, optimizes neurogenesis and synaptic plasticity during early development and throughout the lifespan [14,15]. EPA and DPA both have anti-inflammatory benefits in encephalic tissues [9,16,17] and contribute to the structural integrity of neurons by being converted into DHA [18,19]. DHA in particular, but n3-LCPUFAs in general, are so critical to the brain’s integrity that vertebrates have evolved mechanisms that preferentially transfer DHA to the neuronal tissues of developing offspring through placental transfer, yolk deposition or lactation [20–23].

n3-PUFAs are essential nutrients in vertebrates, yet their availability differs greatly between terrestrial and aquatic ecosystems [24–26]. Terrestrial primary producers are generally incapable of producing n3-LCPUFAs but are rich in ALA [27–29]. As a result, vertebrates that consume terrestrial plants have the necessary enzymes to bioconvert ALA into n3-LCPUFAs through a metabolically expensive process that can meet their structural and metabolic needs [30,31]. By contrast, aquatic primary producers readily synthesize n3-LCPUFAs which bioaccumulate in zooplankton, small fishes and higher-order trophic levels [32,33]. Owing to the abundance of n3-LCPUFAs in aquatic ecosystems, aquatic consumers are generally thought to be unable to synthesize n3-LCPUFAs and must rely instead on dietary consumption to meet their nutritional requirements [34,35].

In urban environments, anthropogenic foods available to animals tend to be deficient in all types of n3-PUFAs but rich in omega-6 polyunsaturated fatty acids (n6-PUFAs) [8,35] owing to the fatty acid profile of major agricultural crops (e.g. soybean, corn and sunflower) at the base of Western diets [36]. Although n6-PUFAs are also essential to vertebrates, notably for their role in immunity and their contribution to neuronal tissues [37–39], their abundance in human-made foods can lead to adverse health effects if not counterbalanced with an equally high consumption of n3-PUFAs [8]. n6-PUFAs are proinflammatory compounds because they produce acute inflammation in response to injury or illness [40]. While inflammation is an integral part of healing, it must be counterbalanced by anti-inflammatory agents, such as n3-LCPUFAs, which protect tissues from long-term damage caused by oxidative stress [16,41,42]. In addition, foraging in cities and landfills is, in itself, proinflammatory owing to the heightened oxidative stress experienced by urban populations as a result of greater exposure to pollution and contaminants [6,43,44]. The combination of foraging in habitats conducive to oxidative stress and feeding on resources high in proinflammatory n6-PUFAs but poor in anti-inflammatory n3-LCPUFAs put urban animals at greater risk of suffering adverse consequences from long-term inflammation, whether it be through impaired fertility [45,46], reduced longevity [47,48] or early onset of brain senescence [15,49,50]. Maintaining a balanced ratio of n6- to n3-PUFAs is thus essential to combat long-term inflammation, especially because n6-PUFAs compete metabolically with n3-PUFAs for absorption and use in tissues [39,51,52]. An ideal n6- to n3-PUFA ratio for humans was determined to be below 4 : 1 [8] but this ratio is probably species specific [53].

In humans and rodents, omega-3 fatty acids must be consumed throughout life because they are continuously metabolized in the brain [54,55]. In fact, adult mammals (and fishes [56]) that feed on an aquatic diet tend to accumulate more n3-LCPUFAs in their brains compared with conspecifics consuming a Western-like diet [56–58]. Low intake of n3-LCPUFAs in adulthood can damage the structural integrity of the brain and lead to losses in grey matter volume [59,60], yet, these losses can be stopped and even mitigated by the renewed intake of n3-LCPUFAs [61–63].

Consuming n3-LCPUFAs has been shown repeatedly to benefit brain health in mammals, yet little is known about its importance in maintaining or optimizing the brain’s integrity and function in other taxonomic groups such as birds. Only one study has tested whether the fatty acid composition of avian neuronal tissues remains sensitive to diet beyond the nestling stage [64]. The authors successfully increased the concentration of n3-LCPUFAs in the brains of captive adult zebra finches (*Taeniopygia guttata*) through dietary supplementation, which suggests that the fatty acid composition of the avian brain might, like the mammalian brain, remain plastic during adulthood [64]. Since perching birds such as zebra finches are well-known for brain plasticity in adulthood [65–67], it is perhaps not surprising that their encephalic fatty acid profile can reflect their immediate diet as their brains undergo acute neurogenesis each year [68–70]. By contrast, we are not aware of any studies that have examined the encephalic fatty acid profile of the non-passerine adult avian brain or explored its sensitivity to an individual’s recent diet.

In this study, we tested whether recent or seasonal dietary changes explain the fatty acid content of the brains of wild adult ring-billed gulls (*Larus delawarensis*). This non-passerine species is ideal for this study because their diet can range from primarily anthropogenic food to primarily marine resources [71,72]. Owing to their generalist foraging behaviour, many species of gulls (*Larus* spp.) have been successful at exploiting human-made food, often favouring anthropogenic resources even in situations where their natural aquatic prey remain accessible (e.g. herring gulls, *Larus argentatus* [73]; yellow-legged gulls, *Larus michahellis* [74]; ring-billed gulls [72] and lesser black-backed gulls, *Larus fuscus* [75]). Often, heightened reliance on rubbish has been associated with increased fitness, with landfill and urban foraging correlating with increased population size [76], clutch size, egg mass [77], fledging success [78] and adult body condition [77,79]. Nonetheless, replacing aquatic diets with anthropogenic diets has also been linked to adverse outcomes, including declining population density despite gulls laying larger eggs [80], reduced brood size [81], lower nestling body mass [82] and decreased long-term reproductive success [83]. Foraging on rubbish and at landfills is also linked to greater exposure to heavy metals [84,85], contaminants like flame-retardants [86–88], pathogens ([89,90] but see [91]) and harmful non-digestible items such as plastic and broken glass [92,93], which could all lead to adverse reproductive success or survival [94–97].

We have previously demonstrated that the cerebral fatty acid profile of ring-billed gull nestlings responds to short-term dietary supplementation, though it remains unknown whether this brain plasticity persists through adulthood [98]. Here, we focused on adults, using an urban breeding colony foraging mainly on anthropogenic foods and a more natural-like breeding colony foraging primarily on marine organisms. We used the combination of fatty acid signatures and stable isotope biomarkers to understand, at the individual scale, the short- and long-term diets of gulls nesting at both sites [99,100]. We also attempted to increase the n3-LCPUFA content of the brains of urban nesters by supplementing them with fish oil during incubation. Concurrently, we supplemented natural nesters with coconut oil in an attempt to reduce their consumption of marine food and thus reduce the n3-LCPUFA levels of their brains. For each individual, we determined whether their colony’s normal diet and the type of supplementation they received was reflected in the fatty acid composition of their brain. Since gulls’ diets can change drastically outside the breeding season [101–103], we also analysed the stable isotope signatures of feathers grown at different times of the year to determine whether the n3-LCPUFA profile of their brains was best predicted by their most recent diet or by long term dietary specialization.

## 2. Material and methods

### 2.1. Ethical statement

All methods were performed under appropriate permits (Canadian Wildlife Service Scientific Permit, number SC4049; Environment and Climate Change Canada Scientific Permit to Capture and Band Migratory Birds, numbers 10 890 and 10 890B) and were approved by Memorial University of Newfoundland and Labrador’s Animal Care Committee (number 19-03-DW).

### 2.2. Study sites and subjects

**Figure 1.**
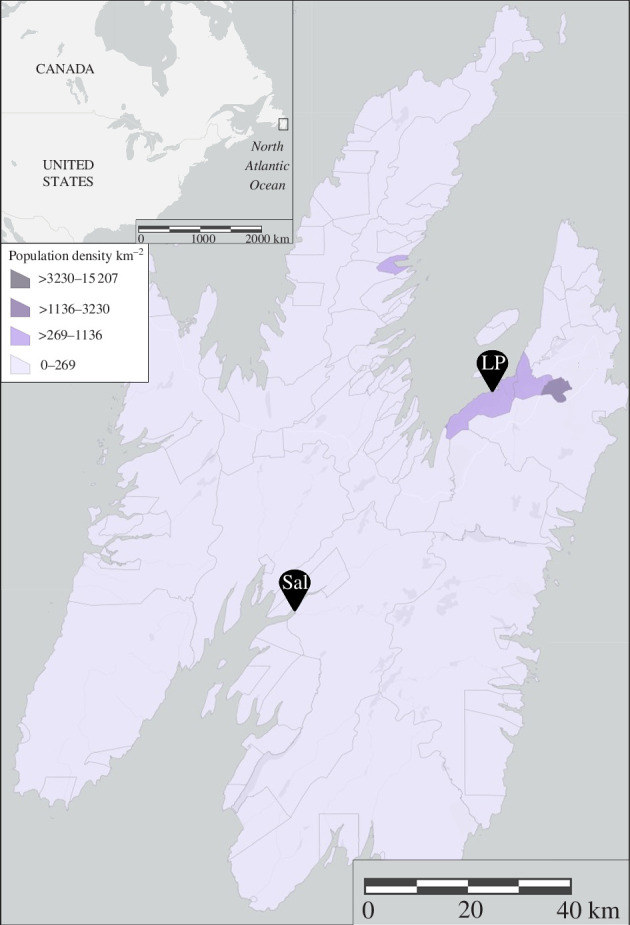
Locations of the two ring-billed gull colonies studied on the coastline of the island of Newfoundland, Canada in 2021 in relation to the human population density (number of people km^−2^) of the same year [104]. The Long Pond colony (LP; 47°31′09.8′′ N, 52°58′33.6′′ W) is situated in an urban environment whereas the Salmonier colony (Sal; 47°08′11.0′′ N, 53°28′48.6′′ W) is situated in a natural environment.

From 13 May to 18 June 2021, we visited two breeding colonies of ring-billed gulls daily throughout their incubation period. Both colonies were situated along the coastline of the island of Newfoundland, Canada (figure 1). Although both colonies are located on sandbars bordered by the Atlantic Ocean, the Long Pond colony is situated in an urban environment where terrestrial and anthropogenic food abound, whereas the Salmonier colony is situated in a more natural environment where marine organisms are the main food resources. We have previously shown that these two colonies are on opposite sides of the dietary spectrum during incubation, with birds nesting at Long Pond feeding mainly on anthropogenic and terrestrial resources deficient in n3-LCPUFAs and birds nesting at Salmonier feeding mainly on marine organisms rich in n3-LCPUFAs [72].

**Figure 2.**
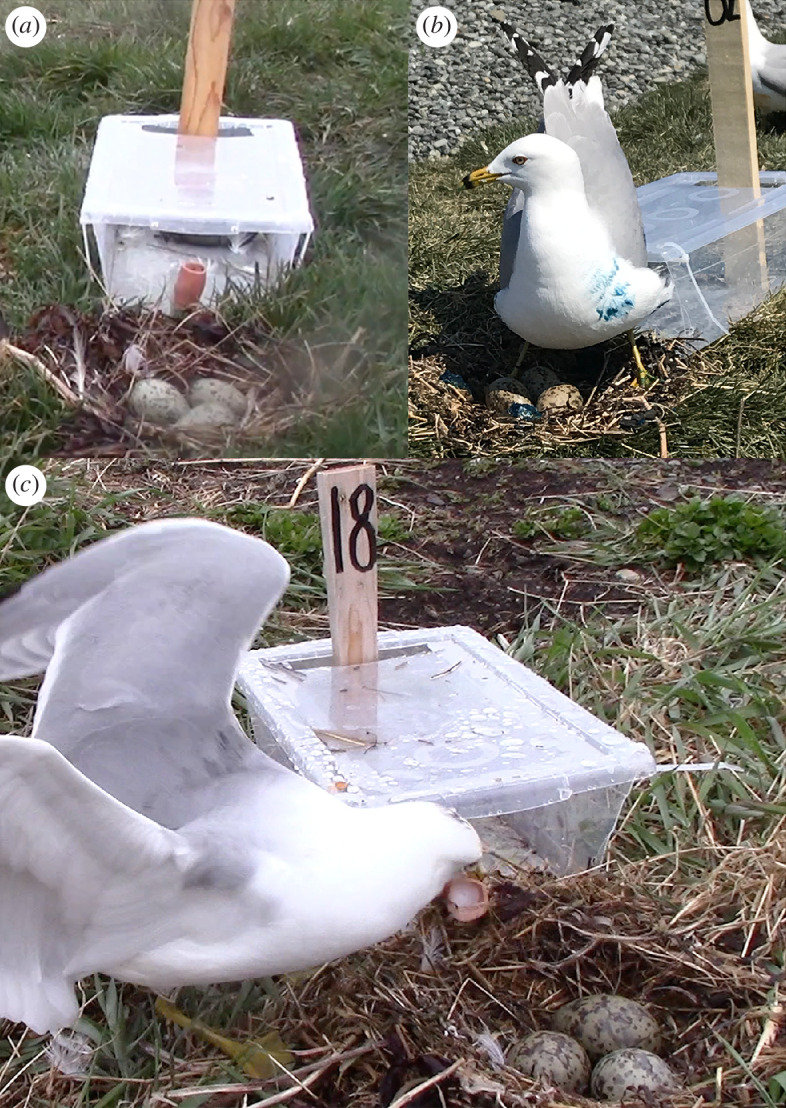
An empty puzzle box was staked with a numbered post beside each target nest and used to identify each nest to its treatment group and to deliver the supplement. (*a*) Hollowed-out sausage containing the fish oil supplement left at the rim of the empty puzzle box. (*b*) Gull marked with colourful dye, making them easily recognizable as the parent eating the intended supplement left at their nest. (*c*) Parent collects a coconut oil supplement from the puzzle box placed beside its nest. Note the small black dye mark on the top of the gull’s head, which was used to identify individual gulls during the final week of supplementation and during subsequent cognitive testing that occurred as part of another study.

At the start of the laying period of the Long Pond colony, we randomly assigned 30 nests with partially completed clutches (i.e. one to two eggs per nest; a typical nest has three eggs [105]) to each of three supplemental feeding treatments (i.e. *n* = 90 nests): an experimental treatment in which subjects were supplemented daily throughout incubation (22 days) with fish oil rich in n3-LCPUFAs, a positive control treatment in which subjects were supplemented daily with coconut oil devoid of n3-LCPUFAs, and a negative control treatment in which subjects were not supplemented. Concurrently, at the start of the laying period of the Salmonier colony, we randomly assigned 30 nests to an experimental group where subjects were supplemented daily with coconut oil and 30 nests to a negative control group where subjects were not supplemented (i.e. *n* = 60 nests). We excluded the fish oil treatment because Salmonier nesters already consume an exclusively marine diet during the breeding season [72], such that it would not be ecologically relevant to increase n3-LCPUFA consumption beyond that point. Instead, we used the negative control group at the Salmonier colony to define the natural ceiling of n3-LCPUFAs stored in tissues and to determine whether that concentration could be reduced by providing the birds with a caloric substitute devoid of n3-LCPUFAs (experimental group). Therefore, the coconut oil served as a positive control for the Long Pond colony because it was not expected to alter the n3-LCPUFA consumption of urban nesters, whereas it served as an experimental treatment for the Salmonier colony because it was expected to lower the natural nesters’ n3-LCPUFA intake. Each target nest (*n* = 150) was marked by placing an empty puzzle box next to it and staking the box to the ground with a numbered post (figure 2). We used the puzzle box for another study investigating the problem-solving skills of these birds. The parents were also passively marked with colourful dyes during the final week of supplementation as part of the cognitive tests following the methods described in [106].

### 2.3. Supplementation

The daily supplement was embedded in a hollowed-out sausage and placed on the floor of the puzzle box along the edge closest to the nest (figure 2). Placing the supplement inside the box helped to protect it from nearby thieves and increased the likelihood that the parents were the ones consuming the supplement. Supplements were delivered within 45 min to all marked nests at a colony in approximately the same sequence each day. The parents flushed briefly from their nests when we were within approximately 1 m but typically returned and resumed incubating within seconds of our departure. Parental absence from the nests was usually brief enough to keep thieves away, though some thievery did occur. In an attempt to limit our time on the colonies to minimize disruption, we did not systematically monitor nests to ensure that the supplements were always consumed by the intended parents. Nevertheless, we anecdotally observed the target gulls consuming their intended supplements during every supplementation bout at both colonies. We could identify the parents of a supplemented nest because they would resume incubation at the same nest immediately after consuming the supplement. By contrast, thieves would consume the supplement and then quickly and immediately move to a nearby nest to resume incubation. During the final week of supplementation, when gulls were passively marked with dye in preparation for cognitive testing, we were able to use the individually distinctive dye marks to further distinguish targeted parents from thieves (figure 2). Based on all of our anecdotal observations throughout the supplementation period, we observed approximately one instance of thievery for every 20 instances of the target gull consuming the supplement.

We determined the size of the daily supplement by calculating the birds’ energetic requirements based on the field metabolic rate equation formulated for seabirds by Ellis & Gabrielsen [1,107]:


$$field\;metabolic\;rate=9.014\;mass^{0.655}\times {\left[exp10\left(latitude\right)\right]}^{0.0048},$$

where field metabolic rate is expressed as kJ d^−1^ and body mass is expressed in g.

We used a mass of 468 g, which was the average mass of ring-billed gulls nesting at the Long Pond and Salmonier colonies in 2020 [72]. The latitude of both colonies is 47° N, and the field metabolic rate, therefore, was calculated to be 850.21 kJ d^−1^. We then calculated the amount of n3-LCPUFAs that gulls would ingest daily on an exclusively piscivorous diet. Assuming a diet comprising 850.21 kJ d^−1^ of capelin (*Mallotus villosus*), this equated to 1.49 g n3-LCPUFAs d^−1^ [108]. Since ring-billed gulls provide biparental care and split their incubation duty evenly between mates [105], we attempted to supplement both parents of each target nest equally by alternating the time at which the supplementation was given (early morning or late afternoon) on a daily basis. Based on our experience working with these colonies the previous year [72], incubation shift change could happen at any time of the day and the same mate was not consistently at the nest at the same time every day. As a result, we chose to alternate the supplementation time between mornings and afternoons in the hopes of consistently supplementing the largest number of parents possible, although some mates might have received the bulk of the supplementation left at their nest while others got little or none of it.

Working from the hypothesis that each mate would receive the supplementation every other day, we also nearly doubled the n3-LCPUFA dose given daily (2.88 g) to ensure that each parent received its maximum daily dose on average. The 0.10 g discrepancy between the calculated supplement size (2.98 g n3-LCPUFA) and the actual size of the daily supplement (2.88 g n3-LCPUFA) was because we used pre-made fish oil capsules as our supplements to prevent oxidation and to ensure the ingestion of the whole n3-LCPUFA dose. We, therefore, could not adjust the size of the capsules.

The fish oil supplement included three fish oil capsules (Webber Naturals^TM^ triple-strength Omega-3 softgels) embedded in a hollowed-out sausage. The three capsules together contained 4275 mg of fish oil (table 1; 161 kJ) providing 1781.77 mg EPA, 191.22 mg DPA and 906.47 mg DHA, as well as 956.87 mg of other fatty acids (see table 1). The coconut oil supplement included a caloric equivalent of coconut oil (Kirkland Signature^TM^ Organic Virgin Coconut Oil; 4.27 g, 161 kJ), also embedded in a hollowed-out sausage. The coconut oil supplement included 3930.64 mg of fatty acids devoid of n3-LCPUFAs (table 1). The negative control groups did not receive any dietary supplement or sausage, but we performed a sham action of leaving a supplement at their nest to standardize the level of disturbance caused by the investigators across all target nests.

**Table 1.** Fatty acid composition, expressed as the percentage of total identified fatty acids, of the fish oil and coconut oil supplements given daily to ring-billed gulls during their incubation period, as well as the composition of the hollowed-out sausage (chicken meat) used as an edible carrier for the supplements. (Trace indicates that the concentration of the fatty acid was below 0.01%. n.d. indicates that the fatty acid was not detected. LA, linoleic acid; ALA, alpha-linolenic acid; AA, arachidonic acid; EPA, eicosapentaenoic acid; DPA, docosapentaenoic acid; DHA, docosahexaenoic acid).

fatty acid	**c**oconut oil (%)	**f**ish oil capsule (%)	**s**ausage (%)
C10:0	4.64	trace	trace
C11:0	0.03	trace	trace
C12:0	55.02	trace	trace
C13:0	0.04	trace	trace
C14:0	23.49	0.10	2.90
C14:1	trace	trace	0.02
C16:0	11.23	trace	22.67
C16:1 *n*−11	n.d.	0.02	0.00
C16:1 *n*−9	0.01	1.24	0.99
C16:1 *n*−7	trace	0.01	3.08
C16:1 *n*−5	trace	0.02	0.02
C16:2 *n*−6	trace	1.09	0.02
C17:0	0.01	trace	0.20
C16:3 *n*−4	trace	1.24	0.23
C16:4 *n*−3	trace	0.08	0.02
C16:4 *n*−1	trace	2.40	0.02
C18:0	4.27	0.02	25.42
C18:1 *n*−11	n.d.	1.71	0.11
C18:1 *n*−9	0.06	0.15	26.88
C18:1 *n*−7	trace	trace	3.54
C18:1 *n*−6	trace	0.02	n.d.
C18:1 *n*−5	trace	0.01	n.d.
C18:2 *n*−6 (LA)	0.96	0.99	9.83
C18:2 *n*−4	trace	0.35	0.03
C18:3 *n*−4	trace	0.33	0.06
C18:3 *n*−3 (ALA)	trace	0.74	1.19
C18:4 *n*−3	trace	6.85	0.02
C18:4 *n*−1	n.d.	0.64	trace
C20:0	0.12	0.12	0.19
C20:1 *n*−11	0.05	0.19	0.04
C20:1 *n*−9	trace	0.14	0.14
C20:1 *n*−7	trace	0.01	trace
C20:2	0.01	0.02	n.d.
C20:2 *n*−6	trace	0.15	0.14
C20:3 *n*−6	n.d.	0.25	trace
C20:4 *n*−6 (AA)	n.d.	2.40	2.00
C20:3 *n*−3	trace	0.11	trace
C20:4 *n*−3	n.d.	1.85	trace
C20:5 *n*−3 (EPA)	trace	46.31	trace
C22:0	0.02	0.21	trace
C22:1 *n*−9	n.d.	0.09	trace
C22:1 *n*−7	trace	0.09	trace
C22:2 *n*−6	trace	0.03	trace
C22:4 n−6	n.d.	0.37	trace
C22:3 *n*−3	n.d.	0.02	trace
C22:5 *n*−6	trace	0.68	trace
C22:4 *n*−3	trace	0.13	trace
C22:5 *n*−3 (DPA)	trace	4.97	trace
C22:6 *n*−3 (DHA)	trace	23.56	n.d.
Σ SFAs^a^	98.86	0.45	28.54
Σ MUFAs^b^	0.12	3.69	34.82
Σ PUFAs^c^	0.96	95.56	13.57
Σ *n*−6 FAs^d^	0.96	4.87	11.97
Σ *n*−3 FAs^e^	trace	84.39	1.22
Σ *n*−3 LC FAs^f^	trace	74.84	trace

^a^ Sum of saturated fatty acids: C10:0+C11:0+C12:0+C13:0+C14:0+C16:0+C17:0+C18:0+C20:0+C22:0.

^b^ Sum of monounsaturated fatty acids: C14:1+C16:1 *n*−11+C16:1 *n*−9+C16:1 *n*−7+C16:1 *n*−5+C18:1 *n*−11+C18:1 n−9+C18:1 *n*−7+C18:1 *n*−6+C18:1 *n*−5+C20:1 *n*−11+C20:1 *n*−9+C20:1 *n*−7+C22:1 *n*−9+C22:1 *n*−7.

^c^ Sum of polyunsaturated fatty acids: C16:2 *n*−6+C16:3 *n*−4+C16:4 *n*−3+C16:4 *n*−1+C18:2 *n*−6+C18:2 *n*−4+C18:3 *n*−4+C18:3 *n*−3+C18:4 *n*−3+C18:4 *n*−1+C20:2+C20:2 *n*−6+C20:3 *n*−6+C20:4 *n*−6+C20:3 *n*−3+C20:4 *n*−3+C20:5 *n*−3+C22:2 *n*−6+C22:4 *n*−6+C22:3 *n*−3+C22:5 *n*−6+C22:4 *n*−3+C22:5 *n*−3+C22:6 *n*−3.

^d^ Sum of omega-6 polyunsaturated fatty acids: C18:2 *n*−6+C20:2 *n*−6+C20:3 *n*−6+C20:4 *n*−6+C22:2 *n*−6+C22:4 *n*−6+C22:5 *n*−6.

^e^ Sum of omega−3 polyunsaturated fatty acids: C18:3 n−3+C18:4 *n*−3+C20:3 *n*−3+C20:4 *n*−3+C20:5 *n*−3+C22:5 *n*−3+C22:6 *n*−3.

^f^ Sum of long-chain omega−3 polyunsaturated fatty acids: C20:5 *n*−3+C22:5 *n*−3+C22:6 *n*−3.

The hollowed-out sausage was used as an edible carrier to hold the supplements upright when placed in the box by the gulls’ nests (approximately 10 g of sausage used per supplement; figure 2). We purchased house-brand chicken sausages devoid of n3-LCPUFAs (table 1), which have been successfully recognized as a rewarding food item in the past by the gulls nesting at those same colonies [72,109]. The sausages stuffed with the fish oil capsules or the coconut oil were kept on ice until they could be distributed to the target nests.

### 2.4. Tissue sampling

Following 22 days of supplementation, gulls underwent 3 days of cognitive testing during which they were not supplemented (see Lamarre & Wilson [109] for details of the cognitive testing procedure). We then used noose- and box traps at most target nests to capture as many parents from each treatment group and colony as possible. We weighed captured birds in a cloth bag with a Pesola spring scale (precision: ±5 g), then clipped 1 cm of the tip of two head feathers and 1 cm of the tip of the left and right P1 and P10 primary feathers for use in stable isotope analysis. In ring-billed gulls, head feathers are grown in winter just before spring migration, P1 feathers are grown in summer shortly after the breeding season, and P10 feathers are grown in late autumn, immediately before migration [105]. Since the feathers were collected during the 2021 incubation season, the P1 clippings inform us of the gulls’ diet in summer 2020 following their breeding season, the P10 clippings inform us of their diet during autumn 2020, and the head feathers inform us of their diet during winter 2021. Determining the stable isotope signatures of feathers grown at different time points thus provides a snapshot of their diet at the time of growth [110]. We also used a hypodermic syringe to draw up to 1.2 ml of blood from the brachial vein for fatty acid analysis and stable isotope analysis. The blood was stored on ice in 600 μl lithium-heparin coated tubes (BD Microtainers with plasma separator; BD, Canada, cat. no. B365985) for up to 12 h before being centrifuged at 2000*g* for 4 min to separate the plasma and cell fractions. The plasma phase was transferred into an Eppendorf tube and both plasma and red blood cell (RBC) fractions were stored at −20°C until analysis.

Although we included 150 nests in our study and aimed to capture the parents of as many target nests as possible, we expected to only be able to capture a small subset of our subjects based on previous experience at these colonies. Indeed, the gulls quickly learned to avoid us such that we stopped trapping after 2 days at each colony owing to diminishing catch rates and to minimize disturbance. We were able to capture 33 parents from 29 nests at Long Pond (*n* = 9 in the fish oil group, 11 in the coconut oil group and 13 in the negative control group) and 17 gulls from 15 nests at Salmonier (*n* = 6 in the coconut oil group and 11 in the negative control group). Of all the gulls captured, we randomly selected and euthanized by cervical dislocation one parent from each of eight different nests per treatment group at Long Pond (*n* = 24) and from each of four different nests per treatment group at Salmonier (*n* = 8). We euthanized fewer birds per treatment at Salmonier because our previous research indicated that the fatty acid levels of gulls nesting there were less variable [72]. The carcasses were immediately placed on ice in the field and then stored whole at −20°C within 12 hours of death. They remained stored at −20°C for three months until fatty acid analysis could be undertaken. All other captured birds were banded with a metal Canadian Wildlife Service band on their right leg and an alpha-numeric coded plastic colour band on their left leg before being released. Since ring-billed gulls readily adopt eggs and young chicks but are likely to abandon their young if they lose their mate [111], eggs belonging to sacrificed birds were renested into neighbouring nests containing fewer than three eggs.

### 2.5. Fatty acid analysis

Brain and RBC samples were processed at the Core Research Equipment and Instrument Training Aquatic Research Cluster facility at Memorial University. We dissected the cerebral hemispheres out of the frozen skulls, flash-froze them with liquid nitrogen, then pulverized and homogenized them using a mortar and pestle. Lipids were extracted from 300 μl of the RBC fraction and from 30 mg of the homogenized cerebral hemispheres following methods modified from Folch *et al*. [112]. Modifications included using chloroform, methanol and chloroform-extracted water in a 2 : 1 : 0.5 ratio. The extract was then dried under nitrogen. The fatty acids in the extracted lipids were transmethylated by heating each sample in a mix of 3 ml of Hilditch reagent and 1.5 ml of methylene chloride for 1 h at 100°C. The transmethylation reaction was neutralized by adding 1 ml of saturated sodium bicarbonate solution. The organic phase containing the resulting fatty acid methyl esters was extracted using three hexane washes, and was then dried under nitrogen, reconstituted in 0.5 ml of hexane, and sonicated before undergoing gas chromatography. The fatty acid methyl esters were analysed on an Agilent 7890 gas chromatograph with flame ionization detection and a 7693 autosampler. The gas chromatograph column was a ZB wax+ (Phenomenex, USA; 30 m × 0.32 mm). Fatty acid standards were used (PUFA-1, -3 and Supelco 37 component fatty acid methyl ester mix; Sigma-Aldrich, Canada) to identify the fatty acids by retention time. A quantitative standard (cat. no. GLC490, Nu-Chek Prep, Inc.) was used to check the gas chromatograph column every 300 samples to ensure that the areas returned were as expected. Before transmethylation, an internal standard (nonadecanoic acid C19:0, Sigma-Aldrich, Canada) of known concentration was added to the samples to calculate the concentration of each fatty acid. Results are expressed as relative concentration using percentage of total identified fatty acids.

### 2.6. Stable isotope analysis

In addition to fatty acids, other biomarkers are useful dietary tracers. Specifically, the stable isotope ratios of carbon (^13^C/^12^C, expressed in delta notation as δ^13^C) and nitrogen (^15^N/^14^N, expressed as δ^15^N) found in the tissues of an animal reflects the animal’s diet at the time the tissue was grown [113,114]. Since stable isotopes do not decay over time [115], they are useful for comparing tissues with different turnover rates [110]. For instance, avian RBCs have a turnover rate of 2–4 weeks, therefore, their stable isotope signature reflects their diet over the two to four weeks prior to blood collection [110]. Similarly, because different types of feathers grow at different times of the year following moult, their isotopic profiles reflect the bird’s diet at the time each feather was grown [116]. The bivariate isotopic signature of tissues is shaped by the resources exploited by the animals, where δ^13^C indicates the type of ecosystem in which an animal was foraging and δ^15^N indicates the trophic level from which the resources originate [117,118]. In North America, a diet rich in marine resources produces more enriched δ^13^C values (−24‰ to −19‰) whereas a terrestrial diet is typically more depleted in carbon (<−27‰ [119,120]). However, because the Western anthropogenic diet is rich in tropical plants that use a different pathway to fix CO_2_ (C4 plants instead of the naturally occurring C3 plants of North America), food containing sugarcane or corn (including the livestock that feeds on these plants) tend to be more enriched in carbon (approximately −14‰ [121–123]). For animals with a generalist diet, the δ^15^N signature of their tissues can often distinguish marine foragers (>12‰) from those exploiting anthropogenic resources (<9‰ [74,101,124]). In a previous study, we found that ring-billed gulls nesting at Long Pond had an RBC isotopic signature of −23‰ and 9‰ (δ^13^C and δ^15^N, respectively), which corresponded to their highly terrestrial and anthropogenic diet; Salmonier nesters had a δ^13^C and δ^15^N signature of −20‰ and 13‰, respectively, consistent with a marine diet [72].

The feather samples were prepared for stable isotope analysis following the methods of Chew *et al*. [125]. We first washed the feather samples three times in a 30 : 1 mixture of deionized water and detergent. We then rinsed the samples three times in methanol, three times in methanol : chloroform and three times in chloroform to ensure that all traces of lipids and debris were removed. The feathers were left to air-dry for 48 h afterwards before the barbs were cut into small pieces that would fit into tin capsules. Meanwhile, a 100 μl subsample of each RBC fraction was freeze-dried for 48 h and homogenized. The blood and feather samples were weighed in tin capsules (range of tissue samples: 0.72–1.13 mg) and analysed at the Stable Isotope Laboratory at Memorial University using a Vario Isotope Cube elemental analyser coupled to a Delta V Plus isotope ratio mass spectrometer. The isotope ratios are expressed as parts per thousand (‰) relative to the international standards Vienna Pee Dee Belemnite (VPDB) for *δ* ^13^C and atmospheric N_2_ for *δ* ^15^N following the equation: *δ* ^15^N or *δ* ^13^C = [(*R*_sample_/*R*_standard_) − 1] × 1000, where *R* = ^15^N/^14^N or ^13^C/^12^C, respectively. Ethylenediamine tetraacetic acid (EDTA) no. 2 and USGS62 (both obtained from Indiana University) were used for isotopic calibration. B2155 protein (Elemental Microanalysis) was used as a quality control. Replicates of the quality control (*n* = 4 per run; seven runs in total) indicated overall average s.d. of 0.07‰ for both *δ* ^15^N and *δ* ^13^C, with an accuracy of 0.01‰ for *δ* ^15^N and 0.20‰ for *δ* ^13^C. Owing to the low lipid content of both sample types (C : N_feathers_ = 3.12 ± 0.12 and C : N_RBC_ = 3.43 ± 0.17), lipid extraction was not necessary [126].

### 2.7. Statistical analysis

Analyses were performed in R (version 4.1.0 [127]). For all analyses, we considered n3-LCPUFAs to be the sum of EPA, DPA and DHA. Other long-chain omega-3s were detected in the fish oil supplement (i.e. C20:3*n*3 and C20:4*n*3; table 1), but it is currently unknown whether ring-billed gulls can use them as precursors of EPA since this conversion requires an enzyme that is not present across all vertebrates [128]. For this reason, and because their presence in the fish oil supplement was low in comparison to EPA, DPA and DHA (table 1), they were not included in the calculation of n3-LCPUFAs. Since we did not find any differences between the mass of gulls across colonies or treatment groups, we also did not consider mass in our models.

We validated all parametric models by ensuring that the residuals were normally distributed based on the inspection of quantile-quantile plots and histograms and that homogeneity of variance was met owing to the absence of patterns in the plot of residuals versus fitted values. We also simulated the model’s response and plotted it against the raw data to ensure an adequate overlap. Effect sizes are reported as Cohen’s *d* for *t-*tests and partial *η* ^2^ for ANOVAs and linear models.

For *t*‐test models, the Welch’s test was used when the assumption of homogeneity of variance was violated, otherwise, the Student’s *t*-test was used when the response met the assumptions of normality and homogeneity of variance. In the few cases where ANOVAs were used with the n3-LCPUFA content of RBCs as the response variable, there appeared to be mild departures from normality, though the small sample sizes made it difficult to determine with certainty. We ran those particular analyses using both the parametric ANOVAs and their non-parametric equivalent (Kruskal–Wallis) and, in all cases, results with respect to statistical significance were the same. Therefore, given the limited statistical power associated with non-parametric models and the general robustness of ANOVAs to mild departures from normality [129,130], we opted to use ANOVAs throughout and report only those results, although we provide the raw data in the electronic supplementary material. In all cases, homogeneity of variance was met.

#### 2.7.1. Natural differences in omega-3 long-chain polyunsaturated fatty acid content between colonies

We used Student’s *t*-tests to test whether natural differences existed in the n3-LCPUFA content and the *n*6 : *n*3 ratio of RBCs and brain tissue of gulls breeding at Salmonier versus Long Pond. We focused on the subjects assigned to the negative control groups because their tissues would not have been influenced by supplementation. For analyses using the *n*6 : *n*3 ratio, *n*6-PUFAs refer to the sum of all omega-6s (listed in the electronic supplementary material, tables S1 and S2) that could compete metabolically with any n3-PUFAs (sum of all omega-3s, listed in the electronic supplementary material, tables S1 and S2).

#### 2.7.2. Effect of supplementation on omega-3 long-chain polyunsaturated fatty acid content of red blood cells and cerebral hemispheres

Our second set of analyses tested whether supplementation affected the levels of n3-LCPUFAs or the *n*6 : *n*3 ratio in the RBCs and cerebral hemispheres. We expected the supplements to have different effects based on the gulls' colony. Specifically, we expected the coconut oil treatment to have little effect at Long Pond, where gulls already consume diets deficient in n3-LCPUFAs, and to reduce n3-LCPUFA content at Salmonier where gulls normally consume diets rich in n3-LCPUFAs. Consequently, we also expected the *n*6 : *n*3 ratio of the Salmonier gulls fed coconut oil to increase slightly as a result of decreasing their consumption of marine organisms. We expected the fish oil treatment to increase n3-LCPUFA levels in the tissues of Long Pond gulls. In parallel, we expected the fish oil supplement to decrease the *n*6 : *n*3 ratio in the gulls’ tissues by increasing their levels of n3-LCPUFAs. Since the coconut oil supplement only contained small amounts of n6-PUFAs (table 1), we did not expect a change in the *n*6 : *n*3 profile of the positive control gulls in comparison to their negative control counterparts.

First, we tested the effects of the dietary treatments separately at each colony. At Long Pond, we tested whether dietary treatment (fish oil, coconut oil and negative control) influenced the n3-LCPUFA levels or the *n*6 : *n*3 profile of RBCs and cerebral hemispheres using ANOVAs. When the predictor was found to be significant, multiple pairwise comparisons between treatment groups were investigated and the false discovery rate was controlled using the Benjamini–Hochberg method [131]. At Salmonier, we tested whether dietary treatment (coconut oil and negative control) influenced the *n*6 : *n*3 profile or the levels of n3-LCPUFAs in the RBCs and the cerebral hemispheres using Student’s *t*-tests. We tested the correlation between the levels of n3-LCPUFAs in the RBCs and cerebral hemispheres, as well as the correlation between the *n*6 : *n*3 profiles in the RBCs and cerebral hemispheres, within each colony (all treatment groups combined) using Pearson correlations.

The colony-specific analyses may have lacked statistical power owing to small sample sizes. We, therefore, combined the two colonies in a follow-up analysis. We designed 2 × 2 factorial analyses in which we tested the effects of colony and treatment, plus their two-way interaction, on the n3-LCPUFA content of the gulls’ RBCs and cerebral hemispheres. We repeated the same models using the gulls’ *n*6 : *n*3 profiles as our response variable. The fish oil group at the Long Pond colony (*n* = 9 RBC and eight brains) and the coconut oil group at the Salmonier colony (*n* = 6 RBC and four brains) were categorized as ‘experimental’ and the negative control groups at both colonies remained as negative controls (Long Pond: *n* = 13 RBC and eight brains; Salmonier: *n* = 11 RBC and four brains). We omitted the gulls from the positive control treatment at the Long Pond colony (*n* = 11 RBC and eight brains) because there was no comparable group tested at the Salmonier colony. We expected a main effect of colony (higher n3-LCPUFA levels and lower *n*6 : *n*3 ratio at Salmonier than at Long Pond), no main effect of treatment since the experimental treatments would have opposite effects at the two colonies, and a significant interaction where the experimental treatment would increase the n3-LCPUFA levels (and decrease the *n*6 : *n*3 ratio) of the Long Pond gulls and decrease the n3-LCPUFA levels (and thus increase the *n*6 : *n*3 ratio) in the Salmonier birds. We performed these analyses using linear models followed by pairwise-comparisons adjusted with a Benjamini–Hochberg correction to control for false discovery rate. When modelling the *n*6 : *n*3 ratio response in the gulls’ RBCs, we used general linear models (GLMs) fitted with a Gamma distribution (log link), which provided the best model fit for our positive but skewed data.

#### 2.7.3. Biomarkers of short- and long-term diet as predictors of cerebral omega-3 long-chain polyunsaturated fatty acid content

Our third set of analyses focused on potential seasonal variation in diet. First, we ruled out whether the isotopic signature of the gulls’ RBCs was influenced by their dietary treatment. For Long Pond, ANOVAs were used to compare the *δ* ^13^C and *δ* ^15^N signatures among supplementation groups (fish oil, coconut oil and negative control). At Salmonier, we tested whether the *δ* ^13^C and *δ* ^15^N profiles of RBCs differed between treatment groups (coconut oil or negative control) using Student’s *t*-tests.

We next tested whether the stable isotope signatures of tissues grown at different times of the year indicated that gulls which bred at different colonies maintain distinct trophic niches throughout the year. To do this, we used the *SIBER* package [132] to estimate the isotopic niche breadth of each colony and type of tissue by computing standard ellipse areas corrected for small sample size (SEAc) as well as Bayesian ellipses (SEAb; 10 000 model iterations and the default priors to generate confidence intervals). We then compared the posterior distribution of each SEAb to determine whether the size of its niche breadth was influenced by colony and tissue type. We tested the degree to which each group’s SEAb overlapped with each other when their distributions were plotted on an isotope biplot.

We then tested whether encephalic levels of n3-LCPUFAs during the breeding season were better explained by gulls’ recent diet, as proxied by the fatty acid and stable isotope analyses of RBCs, or by their longer term diet, as proxied by the stable isotope profiles of feathers grown prior to the breeding season. We first explored the correlations among the different isotopic signatures and the gulls’ levels of n3-LCPUFAs in their RBCs to identify potential relationships among predictor variables (electronic supplementary material, figure S1). We detected high collinearity within and among tissues such that using all biomarkers within a single model was not possible. We remedied this issue by performing a principal component analysis based on the correlation matrix. Variables included the δ^13^C and δ^15^N signatures of each tissue, in addition to the level of n3-LCPUFAs in the RBCs. Owing to our small sample size (<100 birds), we applied an orthogonal rotation to the factors (Varimax), as described by Budaev [133]. The first three rotated components had eigenvalues of greater than 1 (electronic supplementary material, table S3 and figure S2) and thus were extracted to be used as covariates in a linear model to test whether the biomarkers of certain tissues grown at certain times of the year explained the level of n3-LCPUFAs in the brains of breeding birds.

## 3. Results

### 3.1. Natural differences in omega-3 long-chain polyunsaturated fatty acid content between colonies

**Figure 3.**
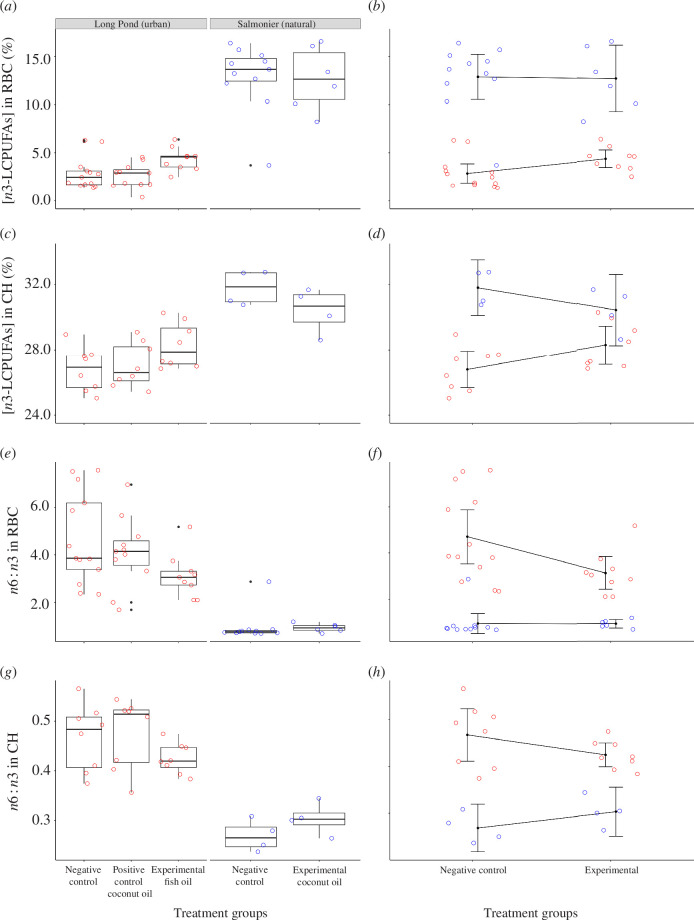
*n*6 : *n*3 profile and n3-LCPUFA content of red blood cells (RBC) and cerebral hemispheres (CH) of ring-billed gulls at the Long Pond and Salmonier colonies after being supplemented daily throughout incubation with fish oil, coconut oil or nothing (i.e. negative control). Raw data are represented by the points, with colours corresponding to the colonies (red = Long Pond and blue = Salmonier). (*a*,*c*,*e* and *g*) Boxplots presenting the differences in the n3-LCPUFA levels (*a*,*c*) or the *n*6 : *n*3 profile (*e* and *g*) of gulls based on their treatment group, colony and tissue. (*b*,*d*,*f* and *h*) Linear model outputs presenting the differences in the n3-LCPUFA content (*b*,*d*) or the *n*6 : *n*3 profile (*f*,*h*) of gulls’ tissues based on whether they received the experimental treatment (fish oil at Long Pond or coconut oil at Salmonier) or were part of a negative control group; Long Pond gulls assigned to the positive control group were excluded from these analyses. Black dots with error bars represent the means ± 95% confidence interval. Concentrations are expressed as percentages relative to total identified fatty acids.

Based on unsupplemented adults from the negative control groups, individuals nesting at the Salmonier colony had significantly more n3-LCPUFAs in their RBCs (mean = 12.88%, s.d. = 3.48%, *n* = 11) than individuals nesting at the Long Pond colony (mean = 2.85%, s.d. = 1.65%, *n* = 13; Student’s *t*-test: *t*_22_ = − 9.25, *p *< 0.001, Cohen’s *d* = 3.79; figure 3). Salmonier nesters also had significantly more n3-LCPUFAs in their cerebral hemispheres (mean = 31.81%, s.d. = 1.07%, *n* = 4) than Long Pond nesters (mean = 26.80%, s.d. = 1.34%, *n* = 8; Student’s *t*-test: *t*_10_ = − 6.45, *p* < 0.001, Cohen’s *d* = 3.95; figure 3).

Accordingly, Salmonier nesters had a lower *n*6 : *n*3 ratio in their RBCs (mean = 1.00, s.d. = 0.65; Welch’s *t*-test: *t*_14.97_ = 6.56, *p *<0.001, Cohen’s *d* = 2.51) and in their cerebral hemispheres (mean = 0.32, s.d. = 0.04; Student’s *t*-test: *t*_10_ = 5.51, *p *< 0.001, Cohen’s *d* = 3.37) compared with their Long Pond counterparts (RBC: mean = 4.95, s.d. = 2.08; cerebral hemispheres: mean = 0.58, s.d. = 0.07; figure 3).

### 3.2. Effect of supplementation on omega-3 long-chain polyunsaturated fatty acid content of red blood cells and cerebral hemispheres

At Long Pond, supplementation had a significant effect on the n3-LCPUFA content of the gulls’ RBCs (ANOVA: *F*_2,30_ = 4.37, *p* = 0.021, *η*_p_^2^ = 0.226; figure 3*a*; electronic supplementary material, table S1). A multiple pairwise comparison revealed that gulls receiving the fish oil supplement had higher levels of n3-LCPUFAs in their RBCs (mean = 4.35%, s.d. = 1.20%, *n* = 9) compared with gulls receiving the coconut oil supplement (mean = 2.59%, s.d. = 1.27%, *n* = 11; *p* = 0.029) or gulls in the negative control group (mean = 2.85%, s.d. = 1.65%, *n* = 13; *p* = 0.032). The coconut oil group did not differ significantly from the negative control group (*p* = 0.650). The n3-LCPUFA content of the cerebral hemispheres did not differ significantly among treatments (ANOVA: *F*_2,21_ = 2.65, *p* = 0.094, *η*_p_^2^ = 0.201), though it showed a similar pattern as for the RBCs (figure 3*c*; electronic supplementary material, table S2).

Supplementation was not a significant predictor of the *n*6 : *n*3 ratio of the Long Pond nesters’ RBCs (ANOVA: *F*_2,30_ = 2.63, *p* = 0.089, *η*_p_^2^ = 0.149) or cerebral tissues (ANOVA: *F*_2,21_ = 1.70, *p* = 0.207, *η*_p_^2^ = 0.139), although trends in the raw data suggested a decreased ratio in the fish oil group (figure 3*e–g*).

At Salmonier, we found no significant effect of supplementation on the n3-LCPUFA content of the gulls’ RBCs (Student’s *t*-test: *t*_15_ = 0.11, *p* = 0.917, Cohen’s *d* = 0.05; figure 3*a*; electronic supplementary material, table S1) or on the *n*6 : *n*3 profile of their RBCs (Student’s *t*-test: *t*_15_ = 0.09, *p* = 0.929, Cohen’s *d* = 0.05; figure 3*e*). Similarly, the supplementation did not significantly influence the n3-LCPUFA levels of the gulls’ cerebral hemispheres (Student’s *t*-test: *t*_6_ = 1.59, *p* = 0.163, Cohen’s *d* = 1.13; electronic supplementary material, table S2) or their cerebral *n*6 : *n*3 ratio (Student’s *t*-test: *t*_6_ = −1.51, *p* = 0.181, Cohen’s *d* = −1.07; figure 3*g*).

When the experimental and negative control gulls from both colonies were combined in the same model to test for an interaction between the colony and supplementation, we found colony to be the sole significant predictor of the n3-LCPUFA content in the gulls’ RBCs (ANOVA: *F*_1,35_ = 95.38, *p* < 0.001, *η*_p_^2^ = 0.788; electronic supplementary material, table S4; figure 3*b*). Specifically, Salmonier gulls had higher n3-LCPUFA levels (mean = 12.82%, s.d. = 3.31%, *n* = 17) than the Long Pond gulls (mean = 3.47%, s.d. = 1.64%, *n* = 22). Neither their supplementation group (ANOVA: *F*_1,35_ = 1.90, *p* = 0.177, *η*_p_^2^ < 0.001) nor the interaction between supplementation and colony (ANOVA: *F*_1,35_ = 1.01, *p* = 0.322, *η*_p_^2^ = 0.006) was statistically significant (electronic supplementary material, table S4; figure 3*b*).

When testing the same predictors’ effects on the *n*6 : *n*3 ratio of the gulls’ RBCs, we found that colony (GLM: LR $\chi_{1}^{2}$ = 63.02, *p *< 0.001; electronic supplementary material, table S4; figure 3*f*) and supplementation (GLM: LR $\chi_{1}^{2}$ = 4.02, *p* = 0.045; electronic supplementary material, table S4; figure 3*f*) were both significant predictors whereas there was no interaction effect detected (GLM: LR $\chi_{1}^{2}$ = 1.54, *p* = 0.214; electronic supplementary material, table S4; figure 3*f*). The Salmonier nesters consistently showed lower *n*6 : *n*3 ratio (mean = 0.95, s.d. = 0.51, *n* = 17) compared with the Long Pond nesters (mean = 4.06, s.d. = 1.76, *n* = 22; figure 3*f*). The gulls that received the experimental treatment had significantly lower *n*6:*n*3 ratio in their RBCs compared with their negative control counterparts (electronic supplementary material, table S4; figure 3*f*).

Colony (ANOVA: *F*_1,20_= 38.0, *p *< 0.001, *η*_p_^2^ = 0.589; figure 3*d*), supplementation (ANOVA: *F*_1,20_ = 4.88, *p* = 0.039, *η*_p_^2^ = 0.014; figure 3*d*), and their two-way interaction (ANOVA: *F*_1,20_= 6.19, *p* = 0.022, *η*_p_^2^ = 0.094) significantly predicted the level of n3-LCPUFAs in the gulls’ cerebral hemispheres (electronic supplementary material, table S4). The significant interaction term between colony and treatment group was further investigated with post hoc tests. The levels of n3-LCPUFAs in the cerebral hemispheres were significantly higher at Salmonier than at Long Pond in both the experimental (*p* = 0.023) and negative control groups (*p *< 0.001). The levels of n3-LCPUFAs in the cerebral hemispheres were also significantly higher among Long Pond gulls that received the fish oil experimental supplement than among Long Pond gulls that received the negative control (Benjamini–Hochberg method: *p* = 0.047; figure 3*d*). By contrast, the levels of n3-LCPUFAs in the cerebral hemispheres did not differ significantly between Salmonier gulls that received the experimental coconut oil and those that received the negative control (Benjamini–Hochberg method: *p* = 0.153; figure 3*d*).

The *n*6 : *n*3 ratio of the gulls’ cerebral tissues was solely predicted by their nesting colony (ANOVA: *F*_1,20_ = 47.32, *p *< 0.001, *η*_p_^2^ = 0.716; electronic supplementary material, table S4; figure 3*h*), where Salmonier nesters showed a lower cerebral ratio of *n*6 : *n*3 (mean = 0.29, s.d. = 0.04, *n* = 8) compared with Long Pond nesters (mean = 0.45, s.d. = 0.06, *n* = 16). Neither the gulls’ supplementation treatment (ANOVA: *F*_1,20_ = 3.25, *p* = 0.087, *η*_p_^2^ = 0.009) nor the interaction between treatment and colony (ANOVA: *F*_1,20_ = 3.58, *p* = 0.073, *η*_p_^2^ = 0.042) predicted the *n*6 : *n*3 ratio of their cerebral hemispheres (electronic supplementary material, table S4; figure 3*h*). The levels of n3-LCPUFAs in the RBCs and cerebral hemispheres were significantly and positively correlated among gulls nesting at Long Pond (Pearson: *r*_22_ = 0.48, *p* = 0.018), but not among gulls nesting at Salmonier (Pearson; *r*_6_ = 0.19, *p* = 0.651). Furthermore, the *n*6 : *n*3 profile of the gulls’ RBCs was positively correlated with the *n*6 : *n*3 profile of their cerebral hemispheres at both Long Pond (Pearson: *r*_22_ = 0.65, *p* < 0.001) and Salmonier (Pearson: *r*_6_ = 0.81, *p* = 0.015).

### 3.3. Biomarkers of short- and long-term diet as predictors of cerebral omega-3 long-chain polyunsaturated fatty acid content

Supplementation did not influence the RBC isotopic signatures of birds nesting at Long Pond (ANOVA; δ^13^C: *F*_2,30_ = 1.54, *p* = 0.232, *η*_p_^2^ = 0.093; δ^15^N: *F*_2,30_= 0.11, *p* = 0.901, *η*_p_^2^ = 0.007) or Salmonier (δ^13^C Student’s *t*-test: *t*_15_ = 0.03, *p* = 0.974, Cohen’s *d* = 0.02; δ^15^N Student’s *t*-test: *t*_15_ = −0.41, *p* = 0.685, Cohen’s *d* = 0.21). The isotopic data from different supplementation groups therefore were pooled in the following analyses.

**Figure 4.**
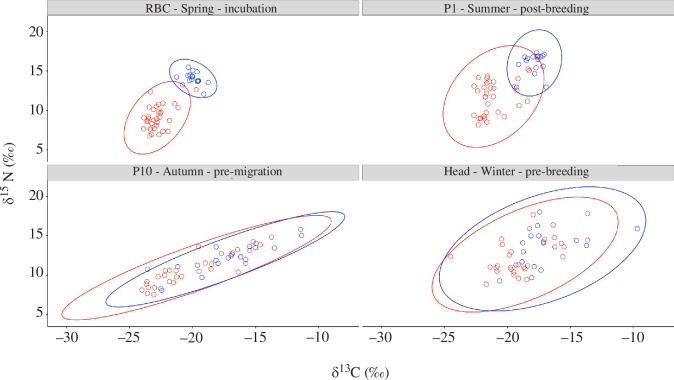
Biplots of the seasonal stable isotope signatures (δ15N and δ13C (‰)) of ring-billed gulls that breed at the urban Long Pond (*n* = 33) and natural Salmonier colonies (*n* = 17). Seasonal diets are inferred from the stable isotope signatures of red blood cells (RBC), which corresponded to the diet during incubation, and the signatures from their feathers, which corresponded to diet post-breeding season (P1, previous year), pre-migration (P10, previous year) and pre-breeding (head). Raw data are represented by the points, with colours corresponding to the colonies (red = Long Pond, blue = Salmonier) and are summarized by their corresponding Bayesian standard ellipse areas (SEAb; 95% credible interval).

**Figure 5.**
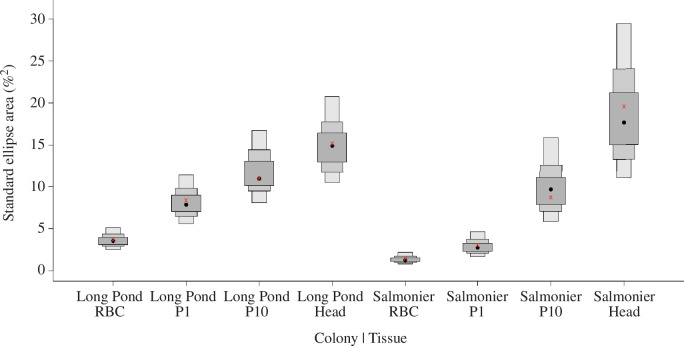
Density plot of Bayesian standard ellipse areas (SEAb) showing the isotopic niche breadths of ring-billed gulls based on their colony (Long Pond and Salmonier) and type of tissue. The tissues represented their diet at the time of growth (RBC = diet during the spring/breeding season; P1 feather = diet during the summer/post-breeding of the previous year; P10 feather = diet during the autumn/pre-migration of the previous year; head feather = diet during the winter/pre-breeding). The black dots correspond to the mode of the SEAb for each group and the red x’s correspond to the mean of the standard ellipse area corrected for small or unequal sample size (SEAc). The light to dark grey boxed areas represent the 95%, 75% and 50% credibility intervals around the SEAb modes, respectively.

We found differences in niche breadth among tissues, both within and between colonies. Gulls from both colonies followed the same pattern, where niche breadth was the narrowest during incubation (RBC signatures), slightly wider in the post-breeding season (based on P1 feathers grown the previous year), wider again before migration (based on P10 feathers grown in autumn of the previous year), and widest during the overwintering period (based on head feathers; figures 4 and 5). Colony comparison of the same tissues indicated that the Long Pond gulls tended to have larger niche breadth throughout the year except during the overwintering period, when Salmonier gulls exploited the largest niche breadth (figures 4 and 5).

The trophic niche of the two colonies overlapped differently at different times of the year. Based on the isotopic signatures of RBCs, the trophic niche of Long Pond gulls did not overlap that of Salmonier gulls during incubation (figure 4). While both colonies had wider trophic niche breadth in the post-breeding season than during incubation, their diets remained distinct (figure 4). Conversely, the isotopic profiles of P10 and head feathers indicated an important overlap between the niche breadths of both colonies during the previous autumn and winter seasons. Compared with their spring and summer signatures, the δ^13^C and δ^15^N signatures of Salmonier nesters were less enriched during autumn and winter whereas those of the Long Pond gulls were more enriched (figure 4).

**Figure 6.**
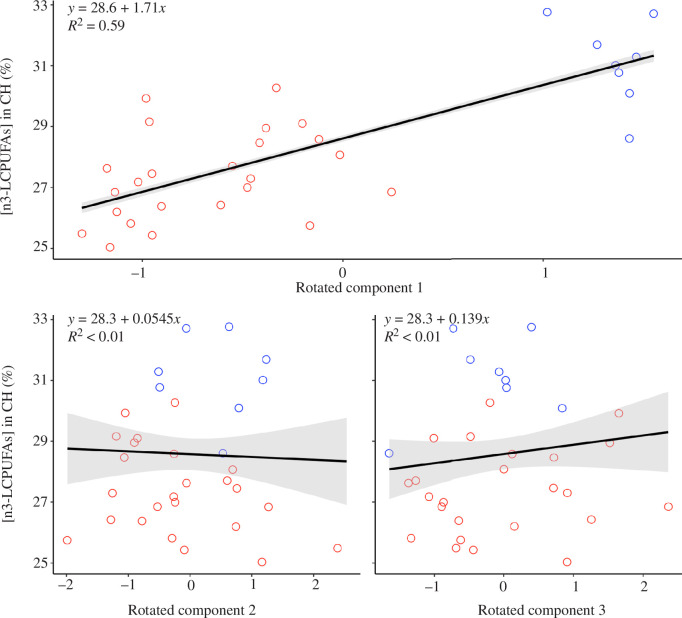
The concentration of n3-LCPUFAs in the cerebral hemispheres (CH) of nesting ring-billed gulls was best predicted by their diet during their recent incubation period and immediately after the previous breeding season. Components 1, 2 and 3 were extracted from a principal component analysis with a Varimax rotation applied. Biomarkers of the gulls’ diet during the breeding season (levels of n3-LCPUFAs and stable isotope signatures of their red blood cells) or immediately after the previous breeding season (isotopic signatures of their P1 feathers) loaded onto component 1. Biomarkers of the gulls’ diet during the previous winter (stable isotope signatures of their head feathers) loaded onto component 2 and biomarkers of the gulls’ diet during the previous autumn (stable isotope signatures of their P10 feathers) loaded onto component 3. The modelled relationships (±95% confidence interval) between the cerebral concentrations of n3-LCPUFAs and each predictor are represented by a black line (with grey shading). Raw data are represented by the points, with the colour corresponding to the colonies (red = Long Pond (*n* = 24), blue = Salmonier (*n* = 8)).

Finally, we tested which tissue biomarkers, and therefore, which seasonal diet, best explained the n3-LCPUFA content of the gulls’ cerebral hemispheres during incubation. The principal component analysis of the δ^13^C and δ^15^N signatures of RBCs and feathers and the n3-LCPUFA levels of RBCs generated three rotated components with eigenvalues of greater than 1.0. Components 1, 2 and 3 explained 49%, 19% and 15% of the variance in the original variables, respectively. The isotopic signatures of RBCs and P1 feathers, as well as the concentration of n3-LCPUFAs in RBCs, loaded positively onto the first component, the isotopic signatures of head feathers loaded positively onto the second component, and the isotopic signatures of P10 feathers loaded positively onto the third component (all loadings ≥ 0.84; electronic supplementary material, table S3 and figure S2). We then used the three components as predictors in a linear model and found that only the first component significantly predicted the gulls’ level of encephalic n3-LCPUFAs (ANOVA: *F*_1,28_ = 45.12, *p* < 0.001, *η*_p_^2^ = 0.594; figure 6). Component two (ANOVA: *F*_1,28_ = 0.13, *p* = 0.718, *η*_p_^2^ = 0.002; figure 6) and component three (ANOVA: *F*_1,28_ = 1.71, *p* = 0.201, *η*_p_^2^ = 0.023; figure 6) were not significant predictors. In other words, gulls with more n3-LCPUFAs in their brains during the breeding season also had more n3-LCPUFAs in their RBCs during the breeding season and more enriched δ^15^N and δ^13^C signatures in their RBCs (produced during the breeding season) and P1 feathers (grown in the summer immediately after the previous breeding season).

## 4. Discussion

The fatty acid composition of adult brains differed between ring-billed gulls nesting at two different colonies, with natural nesters showing greater concentrations of n3-LCPUFAs and accordingly lower *n*6 : *n*3 ratio in their cerebral hemispheres compared with their urban counterparts. We found that the gulls’ diet during the current breeding season or immediately following the previous breeding season best explained the n3-LCPUFA composition of their cerebral hemispheres. Indeed, the fatty acid and isotopic signatures of the Salmonier nesters’ RBCs and P1 feathers indicated a primarily marine diet high in n3-LCPUFAs, which was reflected in the high n3-LCPUFA content of their brains. By contrast, the Long Pond nesters’ biomarkers indicated a mostly anthropogenic or terrestrial diet deficient in n3-LCPUFAs, which coincides with the low n3-LCPUFA levels found in their brains. In addition, we found that the birds’ dietary niches only differed between colonies during the breeding season when they are bound to their colony and shortly after the fledging of their young occurs. During the autumn and the winter, many Salmonier nesters shift from a marine diet towards a more terrestrial or anthropogenic one, and vice versa for the Long Pond nesters. Finally, some of our experimental results also point towards a retained fatty acid plasticity in the brains of adult gulls in response to a short-term dietary change. Individuals supplemented with fish oil at Long Pond incorporated significantly more n3-LCPUFAs into their cerebral hemispheres compared with nesters from the same colony that received no supplementation, even though the fish oil supplementation only lasted 22 days.

Some studies have described population differences in the fatty acid profiles of birds’ RBCs. Their results concord with ours, where urban gulls showed lower concentrations of n3-LCPUFAs and higher *n*6 : *n*3 ratio in their blood than natural gulls during the breeding season [134,135]. However, to our knowledge, no other studies have compared encephalic fatty acid profiles between avian populations. This article provides some of the first cerebral fatty acid data for different populations of wild animals living in urban versus natural habitats. Here, we found the same pattern across the RBCs and brain tissue, namely that our natural nesters fed on a diet high in marine organisms during and immediately after the breeding season and showed a greater accretion of n3-LCPUFAs into their cerebral hemispheres compared with our urban gulls feeding on a mostly anthropogenic diet during the same time frame. The stable isotope signatures of the gulls’ RBCs and feathers indicated a high degree of dietary segregation between the two colonies that only occurred during and immediately after the breeding season; birds from both colonies lost their dietary specialization during the autumn and winter months, as evidenced by their large and overlapping trophic niches. Similar results have been found in other gull species. For example, yellow-legged gulls nesting in a marine habitat tended to exploit a marine diet during their breeding season but shifted towards an anthropogenic diet during their wintering period [136]. By contrast, coastal colonies of yellow-legged gulls and California gulls (*Larus californicus*) nesting in proximity to urban environments had an anthropogenic diet while breeding but favoured marine prey during the winter [74,102]. Like the ring-billed gulls in our study, California gulls increased their niche breadth outside the breeding season [102].

The significant increase in the concentration of n3-LCPUFAs in the RBCs of our Long Pond gulls fed the experimental treatment suggests that the fish oil supplements were consumed by the targeted parents. Furthermore, when we included both colonies in the same statistical analysis, we found evidence that the Long Pond gulls given fish oil incorporated more n3-LCPUFAs into their cerebral hemispheres compared with the negative control group from the same colony. This lends support to the idea that the encephalic fatty acid profile of ring-billed gulls remains plastic in adulthood. These findings are consistent with several mammalian studies which have demonstrated that, in the context of omega-3 dietary deficiency, the introduction of an n3-LCPUFA supplement leads to the rapid accretion of DHA in the subjects’ brain owing to its preferential uptake by encephalic tissue [137–140]. By contrast, we found no evidence that our experimental coconut oil treatment reduced the levels of n3-LCPUFAs in the cerebral hemispheres of the Salmonier nesters. It is possible that our coconut oil supplement did not cause the gulls to reduce their natural intake of marine prey rich in n3-LCPUFAs. Even if the coconut oil did reduce their consumption of marine foods, those gulls may still have consumed enough to allow for the maximum transfer of n3-LCPUFAs into their brains, as seen in rodent models [141,142]. Indeed, the n3-LCPUFA levels of the cerebral hemispheres of Salmonier gulls (range = 28.61–32.76%; electronic supplementary material, table S2) resembled those of exclusively piscivorous vertebrates. For instance, wild salmonids feeding on aquatic organisms had mean DHA levels of 32% in their brain [143], and one-month-old king penguins (*Aptenodytes patagonicus*) had an encephalic fatty acid profile containing 31.5% n3-LCPUFAs [144].

Although our results showed that the n3-LCPUFA content of adult birds’ brains can be altered rapidly through dietary supplementation, this plasticity appears limited as we were only able to increase the cerebral profile of urban nesters from a mean of 26.08% in the negative control group to a mean of 28.27% in the experimental group receiving fish oil (electronic supplementary material, table S2). By contrast, there was a 5% difference between the natural n3-LCPUFA levels in the cerebral hemispheres of our Salmonier (31.81 ± 1.07%) and Long Pond nesters (26.80 ± 1.35%) from the negative control groups. This large natural difference between colonies could have occurred owing to trophic niche segregation occurring around the breeding season. Indeed, considering only the negative control groups, the level of n3-LCPUFAs in the RBCs of Salmonier nesters (12.90 ± 3.48%) was much higher than that of the Long Pond nesters (2.85 ± 1.65%) at the end of incubation. Furthermore, since ring-billed gulls return to their usual nesting sites two to three weeks prior to laying [145], the Salmonier birds would probably have been exploiting an exclusively marine diet for about 1.5 months at the time of brain collection whereas the Long Pond nesters would have been exploiting a diet deficient in n3-LCPUFAs during the same time span.

We cannot exclude the possibility that the 5% difference in cerebral n3-LCPUFAs between colonies is retained from the gulls’ rapid brain development stages (embryogenesis and early life) and that only slight optimization, but no true compensation, can be made to the levels of n3-LCPUFAs in the brain past the juvenile stage. Ring-billed gulls are known to return to the colony where they hatched to breed upon reaching sexual maturity [105], therefore, the *n*6 : *n*3 ratio and the n3-LCPUFA levels observed in the brains of negative control adults may be determined, in part, by the diet they received pre-hatching and pre-fledging. Several studies suggest that the rapid accretion of n3-LCPUFAs in the brain during the late-stage embryogenesis and the first few weeks post-hatch brings the levels of n3-LCPUFAs in the brains of young birds to a level comparable to that of adult birds [11,146,147], implying that brain composition becomes more or less fixed by fledging age. Accordingly, dietary interventions performed on mature poultry and rodents indicate an inability to fully compensate for poor n3-LCPUFA intake during the prenatal or perinatal period, leading to long-term suboptimal levels of n3-LCPUFAs in the brain [14,146,148,149]. During a previous study, we were able to manipulate the n3-LCPUFA levels in the brains of nestling ring-billed gulls [98], but it remains unknown whether those fatty acid levels became fixed upon fledging. The modest increase in the cerebral n3-LCPUFA profile of birds supplemented with fish oil in the current study, therefore, could be explained by a restricted capacity to optimize n3-LCPUFA beyond the levels established prior to maturity.

Among gulls in the negative control groups, the mean concentration of n3-LCPUFAs in the RBCs was 4.5times higher among natural nesters than among urban nesters (electronic supplementary material, table S1). Furthermore, the mean *n*6 : *n*3 ratio in the RBCs was 4.95 among urban nesters versus 1.00 among natural nesters (electronic supplementary material, table S1). The low levels of n3-LCPUFAs and the high *n*6 : *n*3 ratio in the RBCs of urban nesters may place them at increased risk of physiological and cognitive damage. Studies have determined that humans, as terrestrial omnivores, have an overall lower incidence of chronic illness when maintaining their RBC *n*6 : *n*3 ratio under four, but a ratio closer to one is believed to be ideal to successfully prevent long-term inflammation [150]. This ratio might be even less forgiving for marine species that would have historically relied on a highly aquatic diet. This is the case for marine fish where ratios of less than one tend to produce the best outcomes. For example, Atlantic salmon (*Salmo salar*) and Senegalese sole (*Solea senegalensis*) consuming diets with an *n*6 : *n*3 ratio near or below one produce less prostaglandins and other proinflammatory n6-PUFA metabolites as compared with fish fed diets with an *n*6 : *n*3 ratio greater than two [151–153]. Although we showed that some urban nesters switch to a more natural diet during the autumn and winter, which could reduce their *n*6 : *n*3 ratio considerably, their higher risk of oxidative stress would reappear during the breeding season upon resuming anthropogenic foraging. The breeding season is a metabolically demanding time for adults, both in terms of fertility and fecundity but also because of the metabolic cost of providing parental care to eggs and chicks [154–157]. Under such metabolic stress, a high intake of n3-LCPUFAs might increase reproductive success and mitigate reproductive costs among natural nesters, as compared with their urban counterparts. High levels of DHA are required to produce high-quality sperm and eggs [45,46,51,158,159] and high levels of EPA and DPA can be converted into n3-PUFA-derived eicosanoids that actively temper and resolve proinflammatory states [9,17,57,160]. Future research should investigate the fitness consequences of consuming diets with low n3-LCPUFAs and high *n*6 : *n*3 ratios during the breeding season, and whether any such consequences can be mitigated by consuming a more balanced diet during the remainder of the year.

In conclusion, we found two complementary lines of evidence suggesting that the n3-LCPUFA content of a seabird’s brain, the ring-billed gull, remains plastic during adulthood. First, urban and natural nesters had different levels of n3-LCPUFAs in their brains during the breeding season, despite evidence that their diets were similar throughout the autumn and winter. Second, 22 days of fish oil supplementation during incubation was sufficient to influence the brain composition of urban nesters. Longer, more targeted, bouts of supplementation on larger sample sizes are required to determine the sensitivity of the brain to dietary changes, both in a context of n3-LCPUFA deficiency but also under conditions of abundance. Nevertheless, our study is one of the first to suggest that the cerebral levels of n3-LCPUFAs can be manipulated in wild birds through supplementation, despite those birds continuing to consume their typical diet. Future studies should also explore how nesting sites influence the development of nestlings’ brains and whether individuals can fully compensate for an impoverished diet early in life by favouring a diet rich in marine resources post-fledging. Given recent concerns that the levels of n3-LCPUFAs available in food webs will be diminished by an estimated 18–58% by the year 2100 owing to climate change and ocean acidification [161–163], it is becoming imperative to understand how a lack of n3-LCPUFAs might affect the brains and cognition of birds.

## Data Availability

The datasets and R script used in this study are available in the Dryad Digital Repository [164]. Supplementary material is available online [165].
